# A Cheap Battery

**Published:** 1888-08

**Authors:** C. H. Merrick

**Affiliations:** Dwamish, Wash. Ter.


### A Cheap Battery.

To make a cheap battery, get a strip of copper an inch and a half wide and six or eight inches long. To the end, at right angles, solder a piece of insulated copper wire. Coil the copper strip and place it in the bottom of a glass fruit jar; the copper wire will extend three or four inches above the mouth of the jar. Now melt zinc and cast it in any shape so it will enter the mouth of the jar. For example: I frequently take a common spinning top and crowd it into molding sand to form a cup. Pour the cup full of melted zinc and the frustrum of a cone of zinc is formed. To the apex, solder a piece of copper wire; bend the wire over a stick so the zinc will hang in the middle of the jar and about two inches above the coil of copper; the wire from the copper standing up on one side of the jar, the wire from the zinc on the other side.

Put two or three ounces of copper sulphate in the bottom of the jar, with the copper coil; pour in water until it reaches nearly to the apex of the cone of zinc, and one jar of the battery is ready for use. To connect two or more such jars, tie the wire from the zinc of No. 1 to the wire from the copper of No. 2, and so on for as many jars as may be wanted. Two or three such jars will furnish power enough to run two telegraphic sounders with a half mile of wire between them; or they may be used with a vibrator, or as an uninterrupted current. I make my copper strips from bottoms of old boilers or tea kettles. Add copper sulphate to the jars so as to keep the color line of the fluid well above the copper strips but not up to the zinc.

C. H. Merrick, M.D.

Dwamish, Wash. Ter.

---

Methozin is used in the *Medical Standard* as a designation for the drug usually called "antipyrin." Acetanilid is the chemical name of "antifebrin."

Methozin in Epistaxis.—Henocque ("*La Sem. Med.*") has found that the use of a 20 per cent. methozin solution taken by insufflation exerts a very markedly favorable influence on nose-bleed.

